# Editorial: Integration of computational genomics into clinical pharmacogenomic tests: how bioinformatics may help primary care in precision medicine area

**DOI:** 10.3389/fgene.2023.1261876

**Published:** 2023-08-02

**Authors:** Alireza Tafazoli, Mohammad Reza Abbaszadegan, George P. Patrinos

**Affiliations:** ^1^ Department of Analysis and Bioanalysis of Medicines, Faculty of Pharmacy with the Division of Laboratory Medicine, Medical University of Bialystok, Bialystok, Poland; ^2^ Laboratory of Pharmacogenomics, Department of Molecular Neuropharmacology, Maj Institute of Pharmacology Polish Academy of Sciences, Krakow, Poland; ^3^ Department of Medical Genetics and Molecular Medicine, Faculty of Medicine, Mashhad University of Medical Sciences, Mashhad, Iran; ^4^ Medical Genetics Research Center, Mashhad University of Medical Sciences, Mashhad, Iran; ^5^ Laboratory of Pharmacogenomics and Individualized Therapy, Department of Pharmacy, School of Health Sciences, University of Patras, Patras, Greece; ^6^ Department of Genetics and Genomics, College of Medicine and Health Sciences, United Arab Emirates University, Al-Ain, United Arab Emirates; ^7^ Zayed Center for Health Sciences, United Arab Emirates University, Al-Ain, United Arab Emirates

**Keywords:** pharmacogenomics (PGx), clinical bioinformatics, computational genomics and proteomics, statistical genomics, precision medicine

The integration of pharmacogenomic (PGx) tests into daily clinical practice has gained significant momentum in recent years (Arbitrio et al., 2021; Mulder et al., 2021). These tests provide valuable insights into predicting and preventing adverse drug reactions (ADRs) and severe side effects, especially when utilizing a pre-emptive genotyping approach. The advent of high-throughput DNA sequencing technologies has further paved the path for the implementation of personalized medicine. However, there are two primary barriers hindering the rapid integration of advanced sequencing results into clinical reports. The first barrier lies in the vast amount of data generated by these sequencing technologies, necessitating the development of automated approaches for primary and secondary analysis. Bioinformatics plays a critical role in addressing this challenge by providing the necessary tools and algorithms to analyze the obtained data efficiently. Nevertheless, drug-related genes, which are crucial in PGx testing, behave differently from other genes in diseases and require specific attention when employing variant calling tools. The utilization of multiple tools simultaneously has shown promise in overcoming this challenge. The second barrier involves the identification and interpretation of novel variants with unknown clinical significance, often observed through next-generation sequencing platforms. In order to select the most appropriate bioinformatic tools for variant calling, it is essential to have a comprehensive understanding of the available PGx dedicated tools and their main features. Furthermore, common bioinformatics algorithms, such as SIFT, Polyphen2, FATHMM, CAD, etc., may require pre-filtration of variants for specific markers (Tafazoli et al., 2022). However, these tools often overlook variants that result in “increased or decreased function,” which are crucial for determining ultra-rapid and intermediate metabolizer phenotypes.

To address these challenges and promote the widespread acceptance of computational approaches in PGx analysis within clinical settings, this Research Topic focuses on highlighting real-world clinical cases that benefit from bioinformatics tools, algorithms, and other computational and statistic methods. The submitted articles encompass a range of themes, including the role of computational genomics in pharmacogenetics and pharmacogenomics, the advantages of bioinformatics tools and statistical analysis for running clinical pharmacogenomic tests, genomic data management and optimization, and the direct translation of computational analysis of pharmacogenomic test results and genetic-derived therapy into routine clinical practice. Furthermore, the articles also discuss the challenges and future needs in utilizing clinical pharmacogenomic tests as part of daily clinical practice, as well as the attitude and willingness of local pharmacists to train such tests in their routine clinical settings.

Alternative splicing is a fundamental process that plays a crucial role in gene regulation, and its dysregulation has been implicated in various diseases, including cutaneous T-cell lymphomas. In the article of Yu et al., the authors investigate the regulatory effects of alternative splicing (AS) events and RNA binding proteins (RBP) on the efficacy of histone deacetylase inhibitors (HDACi) in treating cutaneous T-cell lymphomas (CTCLs). Through computational data analysis and network mapping, the study identifies specific events and proteins that are associated with HDACi resistance or sensitivity, providing insights into potential targeted treatment strategies for CTCLs (Yu et al.).

As mentioned before, personalized medicine has emerged as a promising approach in healthcare. Understanding community pharmacists’ perceived value, desired training components, and exposure during pharmacy education is essential for assessing their readiness to incorporate this innovative practice into their professional roles and responsibilities. In the article of Naimat et al., the authors explore the value and appeal of precision medicine to community pharmacists in Malaysia. It assesses their knowledge levels, perceptions, and willingness to integrate precision medicine into their daily practice. The study highlights the positive perceptions and high willingness among community pharmacists to adopt precision medicine, emphasizing the need for additional training and exposure to pharmacogenomics during pharmacy education (Naimat et al.).

The investigation on the impact of endogenous and exogenous antioxidants on the risk of multiple cancer types through a Mendelian randomization study, shedding light on the broader role of these compounds in cancer prevention and providing insights into their potential as preventive strategies. In the article of Zhu et al., the authors use Mendelian randomization analysis to assess the causal effect of endogenous and exogenous antioxidants on the risk of six different cancers. The findings reveal potential protective effects of specific antioxidants, such as serum albumin, on prostate cancer risk. The study provides insights into the genetic factors influencing cancer risk and offers potential targeted interventions for cancer prevention, again through the advancement in computational approaches and technologies (Zhu et al.).

Polygenic risk scores in PGx present both promising opportunities and unique challenges. They offer not only a potential avenue for personalized medicine by predicting individual responses to medications based on genetic factors, but also require careful consideration of the complexities involved in their development, interpretation, and implementation within clinical practice. In the article of Simona et al., the authors provide a review on the use of polygenic risk scores (PRS) in PGx. PRS have emerged as a promising tool to account for the complex interplay of genetic factors affecting drug response. The review discusses the general pipeline for PRS calculation and highlights the remaining barriers and challenges in integrating PRS research into clinical care. It emphasizes the need for collaboration between bioinformaticians, treating physicians, and genetic consultants to ensure the transparent and effective implementation of PRS results in real-world medical decisions (Simona et al.). It must be noted, however, that development of PRS is presently only a promising research discipline and under no circumstances can be implemented in clinical reality, not even for clinical decision support, as they clinical evidence is poor and regulatory approval not available.

The studies published in this Research Topic contribute to the advancement of PGx analysis in clinical practice. They provide valuable insights into the potential applications of computational tools and algorithms, the challenges faced in implementing computational approaches, and the future needs in utilizing pharmacogenomics for personalized patient care. By showcasing real-world clinical cases and highlighting the translation of computational analysis into routine practice, these studies will help for the widespread acceptance and integration of pharmacogenomic analysis into healthcare systems worldwide.
